# The Coincidental Evolution of Virulence Partially Explains the Virulence in a Generalist Entomopathogenic

**DOI:** 10.1007/s11686-023-00663-4

**Published:** 2023-02-21

**Authors:** Víctor José Trejo-Meléndez, Texca T. Méndez-López, Jorge Contreras-Garduño

**Affiliations:** 1 Posgrado en Ciencias Biológicas, Universidad Nacional Autónoma de México, Av. Ciudad Universitaria 3000, C.P. 04510, Coyoacán CDMX, Mexico; 2 ENES, Unidad Morelia, UNAM. Antigua Carretera a Pátzcuaro No.8701. Col. Ex-Hacienda San José de la Huerta Código Postal 58190, Morelia, Michoacán Mexico

**Keywords:** Short-sighted evolution, Entomopathogenic nematode, Virulence, Immunomodulation, *Rhabditis regina*

## Abstract

**Purpose:**

The parasites’ virulence is labile after jumping to a new host species, and it might derivate in gaining virulence against a new host as a side effect of living in a non-host environment (coincidental evolution of virulence hypothesis).

**Methods:**

To test this hypothesis, we monitored the experimental evolution of the *Rhabditis regina* nematode for over 290 generations (4 years) in three environments (strains): (1) the natural host, *Phyllophaga polyphylla*, (2) an alternate host, *Tenebrio molitor*, and (3) saprophytic medium (beef; the food that may provide evidence for the coincidental evolution of virulence). Each strain was exposed to *P. polyphylla*, *T. molitor*, or *Galleria mellonella*. We compared the host survival and immune response (proPO, PO, and lytic activity) of infected *versus* uninfected hosts.

**Results:**

The saprophytic nematodes gained virulence only against *G. mellonella.* However, the *P. polyphylla* strain was more effective in killing *P. polyphylla* than *T. molitor*, and the *T. molitor* strain was more effective against *T. molitor* than *P. polyphylla*. Additionally, one dauer larva was sufficient to kill the hosts. Finally, the immune response did not differ between the challenged and control groups.

**Conclusion:**

The coincidental evolution of virulence partially explains our results, but they might also support the short-sighted hypothesis. Additionally, we found evidence for immunomodulation because nematodes passed unnoticed to the immune response. It is crucial to analyze the virulence of entomopathogens from the point of view of the evolution of virulence to be aware of potential scenarios that might limit biological control.

## Introduction

In the host–parasite interaction, parasites evolve virulence strategies that allow them to exploit the host as a food resource, and the host simultaneously evolves resistance through its immune response. This evolutionary arm-race can be analyzed at the molecular and individual level and within or across generations [1, 2]. Since parasites do not always express a high level of virulence, nor do hosts always show an exacerbated immune response against parasites, it is challenging to explore what determines the plasticity of virulence and the host immune response [3, 4]. The scenario of long-term evolution and its genetic basis have been critical aspects of the host–parasite relationship. However, virulence, defined as the reduction in host survival and/or reproduction (host fitness; [5–7]), is labile [8–10], and little is known about the selective pressures that favor or restrict it if parasites jump from one host species to another [11–13]. A relatively untested hypothesis is the coincidental evolution of virulence [14]. This hypothesis poses that the selective pressure exercised by the host is not the only factor involved in virulence. Some biotic and abiotic factors present during the development of the pathogen also influence its virulence in a collateral manner [14]. For example, in bacteria of the genus *Clostridium*, its free lifestyle endows it with characteristics (i.e., toxins) that represent virulence upon confronting a new host, such as humans [14]. Thus, the hypothesis of coincidental evolution predicts virulence against new hosts. In conclusion, the lability of virulence may depend on the parasite's lifestyle; being free, saprophytic, or entomopathogenic, but also on the coincidental evolution of virulence. It is important to note that experimental studies manipulating parasite virulence are key [6]. It is essential to test the coincidental evolution of the virulence hypothesis confronting the parasite against different hosts and with food in which the virulence might be reduced or not needed (i.e. saprophytic food). It is predicted that parasites growing on saprophytic food will be more virulent when they jump into an insect host, in which virulence is important. In addition, the insect host’s jump will result in a reduction of virulence.

The hypothesis of coincidental evolution of virulence might be analyzed by considering host survival and parasite doses as a measure of virulence and the host immune response as it posits a hostile environment for parasites [15]. Furthermore, parasites develop strategies to counteract the immune response of natural hosts [16], which decreases the capacity of the parasite to confront the immune response of new hosts [14, 17]. Parasites may attack the host by manipulating its immune response to diminish or evade (not activate) such response. Making the host immune system less ineffective allows for the successfully establishing the parasite [1, 2, 16]. According to the coincidental evolution hypothesis, parasites can attenuate or avoid activating the immune response of new hosts. Hence, the immune system of new hosts should be weaker in infected than uninfected insects and in infected new hosts versus infected natural hosts.

Entomopathogenic nematodes favor testing the coincidental evolution of virulence hypothesis because they have versatile lifestyles, being free-living, phoretic, necromenic, saprophytic, facultative, or obligate parasites [18]. Moreover, they are known to modulate their host’s immune response [19, 20], whether by evasion or inactivation [21, 22]. For instance, *Heterorhabditis* and *Steinernema* evade the phenoloxidase (PO) cascade of insects and kill their host in days [23–26]. Regarding the innate immune response of insects, the prophenoloxidase system (proPO) is involved in triggering melanization. The proPO activates the phenoloxidase (PO) enzyme, which oxidizes phenols into quinones, and produces melanin and reactive oxygen species (ROS). Melanin is deposited around the nematode, or hemocytes adhere to the parasite and are melanized. In either case, the nematode is encapsulated and isolated from the host body [27], meaning that PO is the central defense of insects against entomopathogenic nematodes and other parasites [28].

The nematode *Rhabditis regina* has been found in Guatemala [29] and Mexico [30, 31]. It infects larvae of *Phyllophaga, Paranomala*, and *Cyclocephala* in its natural environment, but in the lab can infect additional species of insects, such as *Tenebrio molitor*, *Ceratitis capitata* and *Galleria mellonella* [29–31]. Like other nematodes of the *Rhabditis* and *Oscheius* genera, *R. regina* harbors a microbiota composed of entomopathogenic bacteria (i.e., *Serratia* sp. and *Klebsiella* sp. [30, 32–36]. *R. regina* can also develop in a saprophytic medium (e.g., beef), as found with nematodes of the *Pristionchus* genus [37]. The ability of *R. regina* to survive in saprophytic conditions or as an entomopathogen evidences the versatility of its lifestyle. For this reason, the present study aimed to examine whether the variation in virulence of the *R. regina* nematode (Rhabditidae) is explained by the coincidental evolution of virulence. Thus, the evolution of the virulence of the nematode was observed through 290 generations (4 years) in three environments (strains): with a natural host (*P. polyphylla*), with a new host (*T. molitor*), and in saprophytic conditions (beef). Subsequently, each strain was exposed to one of three hosts, simulating a jump of hosts or maintaining the same host infection: *P. polyphylla* or *T. molitor*. We determined the virulence of *R. regina* by considering the hosts' survival according to two doses (one or ten dauer larvae) of nematodes injected per host, and the immune response to know how parasites manipulate the immune responses according to their previous host ambient.

## Materials and Methods

### Strains of *R. regina*

Wild nematodes were obtained from *P. polyphylla* were gathered in cornfields. In the laboratory, the larvae of *P. polyphylla* were monitored for three months to detect the hosts showing signs of sickness by nematodes. The first generation of dauer larvae produced by each dead insect was collected with white traps, and we used all dauer for the experiment [38]. This method was used to collect dauer larvae for the laboratory colonies. The dauer identity was confirmed by submerging the larvae in 1% of with Sodium dodecyl sulfate (SDS; Sigma) for one hour: only the dauer larvae survived this treatment [31]. The strain that infected the natural host in the field (NS) was collected from *P. polyphylla*. The alternative host strain (AS) and the saprophytic strain (beef; SS) were obtained from laboratory-reared colonies: the former breed on *T. molitor* and the latter on cow beef. Nematodes are maintained in chambers (Lumistell) in darkness inside sterilized plates with agar–agar at 26 °C and a relative humidity of 75% for four years, which coincide with about 290 generations because the life cycle of this nematode last about 5 days [29]. A pilot experiment showed virulence changes between these strains after ten generations (unpublished data), so we decided to continue for 290 generations to ensure a change in virulence. We used the SS strain to test the Coincidental Evolution of Virulence hypothesis by simulating a breeding medium not based on insects and hence without the selective pressure due to the immune response. Nematodes can live in this artificial medium for at least ten years [39], and we previously showed that the microbiota changes under this condition compared with nematodes breeding on insects [30].

### Insect Hosts

Given that we were unable to breed *P. polyphylla* in the laboratory, larvae of 3rd instar were collected in the field and used for infections after a quarantine period (after 3 months of no visual showing signs of sickness) to avoid any skew in our results derived to previous infections. Given that the larvae did not feed at this developmental stage, all were maintained individually in small plastic containers of 50 mL with 70% moistened peat moss at room temperature; these conditions were set during the experimental procedure. Larvae of the 12th instar [40] of *T. molitor* were used in infections. Larvae were fed ad libitum with bran and corn meal (3:1), with fresh apple slices added every other day [41, 42]. Finally, we used the 5th instar of *G. mellonella*, which coincide with the size of 1.8 a 1.9 cm [43]. This species was fed ad libitum on a homogenized mix of equal proportions of honey, glycerol, beeswax, dried milk, wheat flour, dry yeast and distilled water, and two servings of corn meal [44.45]. Food was sterilized (125 ± 2 °C for 15 min) to avoid infections [42]. Before inoculating the nematodes, all larvae were topically disinfected with chloride (0.1%). Larvae were caged individually in one well of a six well-plate (Corning), deposited inside an environmental chamber at 27 ± 1 °C and 30% relative humidity in the dark (Lumistell). All infection experiments were carried out in a Purifier Axiom Class II Type C biological safety cabinet (LABCONCO) to avoid contamination, and all material was previously sterilized with UV.

### General Experimental Design

**Fig. 1:**
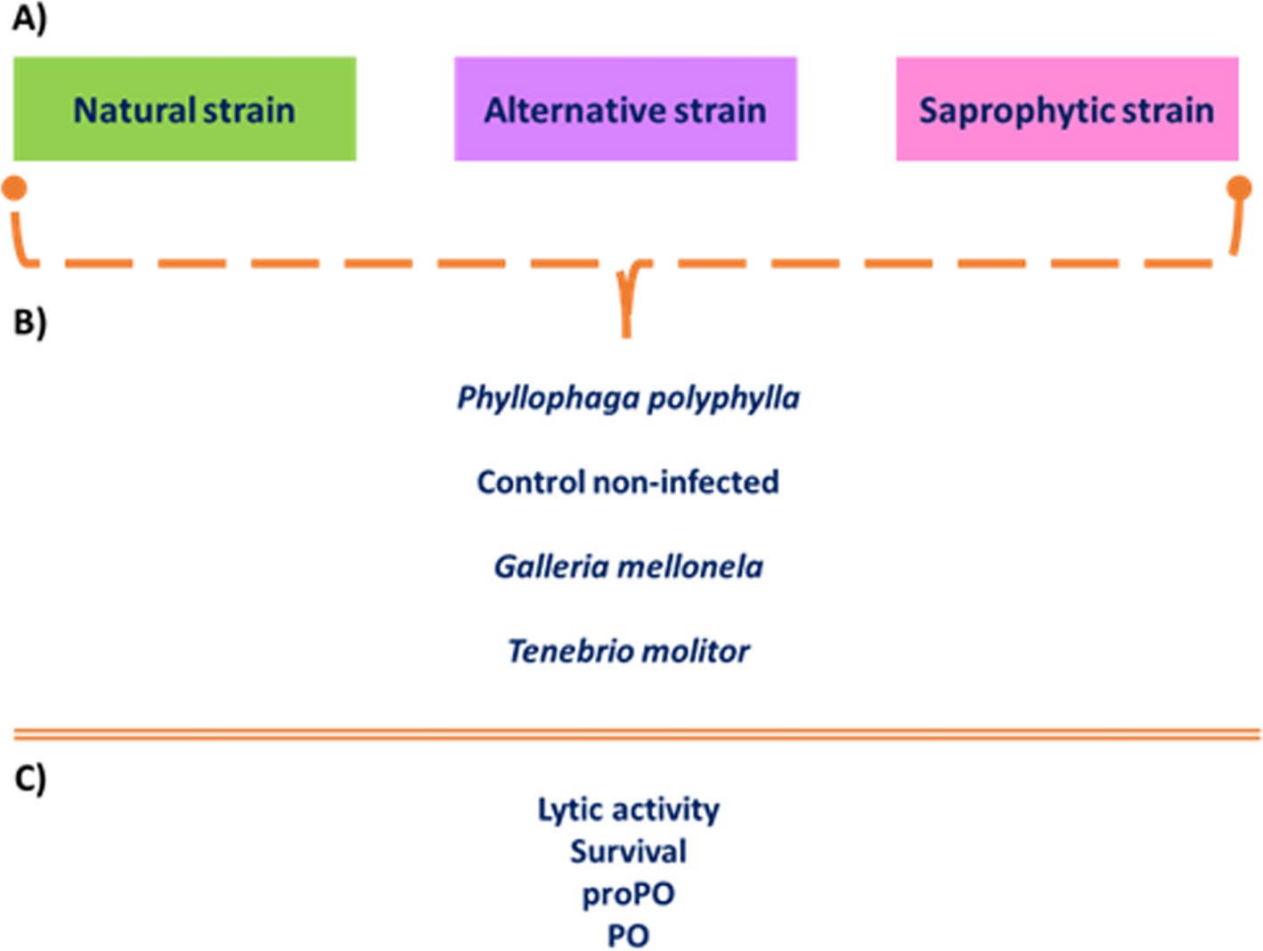
Experimental design to test the coincidental evolution of virulence. **a** We used 3 nematode strains that were breed in each of the following environments: natural strain (*Phyllophaga polyphylla*), alternative strain (*Tenebrio molitor*) or saprophytic medium (beef food). **b** One or ten dauer larvae from each strain was injected into the insect hemocoel being, *P. polyphylla*, *T. molitor* or *G. mellonella*. **c** After 8 h of infection, in one experiment we recorded survival and, in another experiment, we extracted hemolymph to record de immune response (proPO, PO and lytic activity). As a control group, we used non-infected insects from *P. polyphylla*, *T. molitor* or *G. mellonella*

We used a split design [3] using three strains of *R. regina*: a) a strain infecting the natural host *P. polyphylla* (NS), a strain infecting b) an alternative, new host (*T. molitor*; AS), and a strain breed on a saprophytic medium (beef; SS). In each infection (i.e. SS against *P. polyphylla*) we established three sub-groups: a non-infected host or insects infected with 1 or 10 dauer larvae (Fig. 1). Survival and immune response (proPO, PO, and lytic activity) was compared between groups (Fig. 1). This experiment was carried out by triplicate to avoid confounding factors such as genetic drift. They were mixed because no differences were found between replicates (p > 0.05).

### Bioassays of Virulence

We daily recorded survival in the following groups per host (*P. polyphylla, T. molitor*, or *G. mellonella*): the control group received 5 µL a Ringer solution (Sigma) because we diluted the nematodes in this reagent, the 1N or 10 N Groups were injected with 1 or 10 dauer larvae diluted in 5 µL of Ringer. We used micro-syringes (Hamilton 700) to inject the nematodes or Ringer (30 larvae per group). Survival was recorded every day until all infected insects died.

### Immune Response

Hemolymph was extracted 8 h after infection, given that in a previous paper, we demonstrated an effect of time-lapse on immune response after infection [31], and 8 h is conservative. Insects were chilled on ice, and then we made an incision to obtain 4 µL of hemolymph. This 4 µL of hemolymph were obtained with a micropipette (Rainin 10 µL) and deposited in vials of 1.5 mL (Axigen) previously pre-cooled with 200 µL of PBS. Samples were stored at -70 °C in a CryoCube® F570 (Eppendorf) freezer until the analyses of the immune response. Samples were frozen immediately to avoid the activation of the proPO pathway. Additionally, sample collection was split according to experimental groups to avoid any skew in the analyze.

We measured proPO, PO and Lytic activity according to host (Fig. 1). The PO activity was measured spectrophotometrically by recording the formation of dopachrome from L-dihydroxyphenylalanine (L-DOPA, Sigma; [31, 46]). In short, the mixture of hemolymph plus PBS that contained 40 µg/µL of protein was dose-titre until 150 μL with PBS. These 150 µL were mixed with 50 µL of L-Dopa (4 mg/mL [31]). Samples were incubated for 30 min at room temperature inside each 96-well plate (Corning) well and subsequently read in a microplate reader at 490 nm every 5 min for one hour in an ELISA reader (Varioskan Flash Multimode Reader, Thermo Scientific). As blanks, in 3 wells, we mixed 150 µL of PBS and 50 µL of L-DOPA. The enzyme activity was expressed as the rate of change of the optical density in time [31]. To analyze proPO, 5 µL of chymotrypsin (5 mg/mL) were added to the sample (with 40 µg/µL of protein) and then diluted with PBS to reach 150 µl. All these tests were run in the dark. Both PO and proPO were expressed as Activity [31, 47, 48].

Lytic activity was measured in an ELISA plate reader; two wells were used as blanks with 230 μL of PBS. Two other wells received 30 μL of PBS and 200 μL of the suspension of *Micrococcus lysodeikticus* (Sigma). The remainder wells were filled with 30 μL of hemolymph plus PBS and 200 μL of the *M. lysodeikticus* suspension. The suspension of *M. lysodeikticus* was carried out at a concentration of 320 μg/mL, from lyophilized *M. lysodeikticus* powder. After 15 min of incubation at room temperature, the absorbance was read at 540 nm every 5 min for 30. The lytic activity was considered as the degradation of *M. lysodeikticus* according to the linear slope of the absorbance according to time [31]. The more negative slopes denote more activity than the more positive slopes.

### Statistical Analysis

The results were analyzed with the program SPSS Statistics for Windows, Version 22.0 (IBM). A plot of survival of Kaplan–Meier was used, and this was analyzed with a Log-Rank test. For each measurement, the values were fitted to a generalized model with a gamma probability distribution and a logarithmic function after transforming each original variable x to x + 1. This transformation was necessary to use the logarithmic function because of the presence of zeroes in the original variables. Paired comparisons of the means of each dependent variable (proPO, PO and lytic activity) were performed according to the model: strain and host challenge. For each measurement, we fitted the values to a generalized model with a gamma probability distribution and a logarithmic function after transforming each original variable x to x + 1. This transformation was necessary to use the logarithmic function because of the presence of zeroes in the original variables. The effect of the models was tested with a Wald Chi-square test, with a significance threshold of 0.05 and a Bonferroni correction. Mean ± standard errors are reported.

## Results

### Virulence of *R. regina* According to Doses

The log-rank test did not show significant differences between infections with 1 or 10 nematodes of *R. regina* infecting naturally *P. polyphylla* (NS; *X*^*2*^ = 0.03, d.f. = 1, *p *= 0.85), but they differ in *T. molitor* (*X*^*2*^ = 7.36, d.f. = 1, *p* = 0.007) and *G. mellonella* (*X*^*2*^ = 50.35, d.f. = 1, *p* < 0.0001) because a higher mortality was found with 10 than 1 nematode. The AS strain did not reveal significant differences between infections with 1 or 10 nematodes against *P. polyphylla* (*X*^*2*^ = 2.05, d.f. = 1, *p* = 0.15) or *T. molitor* (*X*^*2*^ = 2.05, d.f. = 1, *p* = 0.15), but 10 nematodes killed *G. mellonella* in fewer days than 1 nematode (*X*^*2*^ = 22.93, d.f. = 1, *p* < 0.0001). Finally, the strain SS did not showed differences between 1 or 10 nematodes against *P. polyphylla* (*X*^*2*^ = 0.91, d.f. = 1, *p* = 0.33) nor *T. molitor* (*X*^*2*^ = 1.92, d.f. = 1, *p* = 0.16), but *G. mellonella* in fewer days with 10 nematodes than with only one (*X*^*2*^ = 10.57, d.f. = 1, *p* < 0.001). Due to this result, we only used infection with 10 nematodes in the experiments of immune response.

### Virulence of *R. regina* According to the Host

**Fig. 2:**
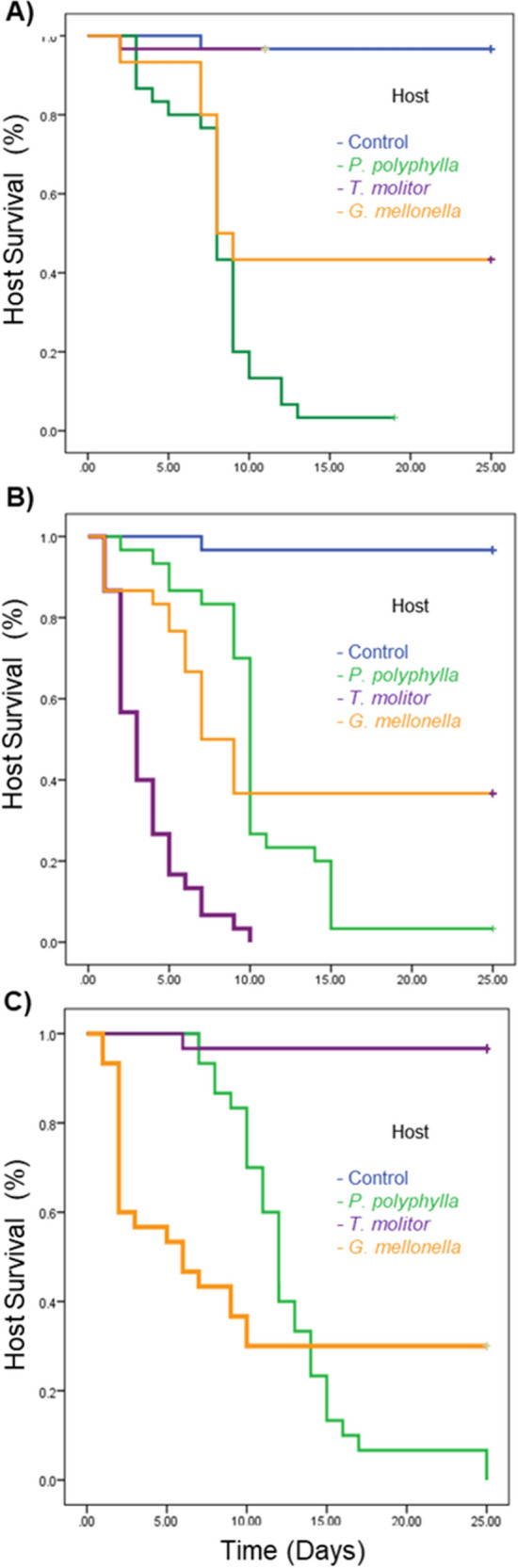
Survival (in days) of insect larvae of *Phyllophaga polyphylla*, *Tenebrio molitor* or *Galleria mellonella* after injecting them with 1 dauer larvae of *R. regina* of the Natural Strain (NS; A), the Alternate Strain (AS; B) or the Saprophytic Strain (SS; C). Some survival lines are overlapped, and it seems that are not shown in the figure

*Tenebrio molitor* did not show differences in survival compared with the control not infected group infected with the *P. polyphylla* natural strain (NS; *X*^*2*^ = 0.00, d.f. = 1, *p* = 0.99), this is the same for *G. mellonella* who showed the same survival as the control group (*X*^*2*^ = 19.594, d.f. = 2, *p* = 0.62) but *P. polyphylla* survived less than *G. mellonella* (*X*^*2*^ = 50.35, d.f. = 1, *p* < 0.01; Fig. 2A). This means that *P. polyphylla* was the more susceptible host against the NS strain.

Considering the alternative nematode strain (AS), the control group was more likely to survive than *P. polyphylla* infected with the strain bred on *T. molitor* (*X*^*2*^ = 43.323, d.f. = 2, *p* < 0.01), followed by *G. mellonella* (*X*^*2*^ = 36.275, d.f. = 1, *p* < 0.01), and *T. molitor* was the more susceptible host (*X*^*2*^ = 44.567, d.f. = 1, *p* < 0.01; Fig. 2B). This revealed that *T. molitor* was the more susceptible host against the AS strain.

Finally, the strain from the beef medium (saprophytic strain; SS) did not kill more the *T. molitor* than the control group (*X*^*2*^ = 1.000, d.f. = 1, *p* = 0.31). *P. polyphylla* survived less than the control not infected group (*X*^*2*^ = 64.65, d.f. = 1, *p *< 0.01) but more than *G. mellonella* (*X*^*2*^ = 14.723, d.f. = 1, *p* < 0.01; Fig. 2C). This suggests that *G. mellonella* was the more susceptible host against the SS strain.

## Immune Response

### proPO Activity

**Fig. 3:**
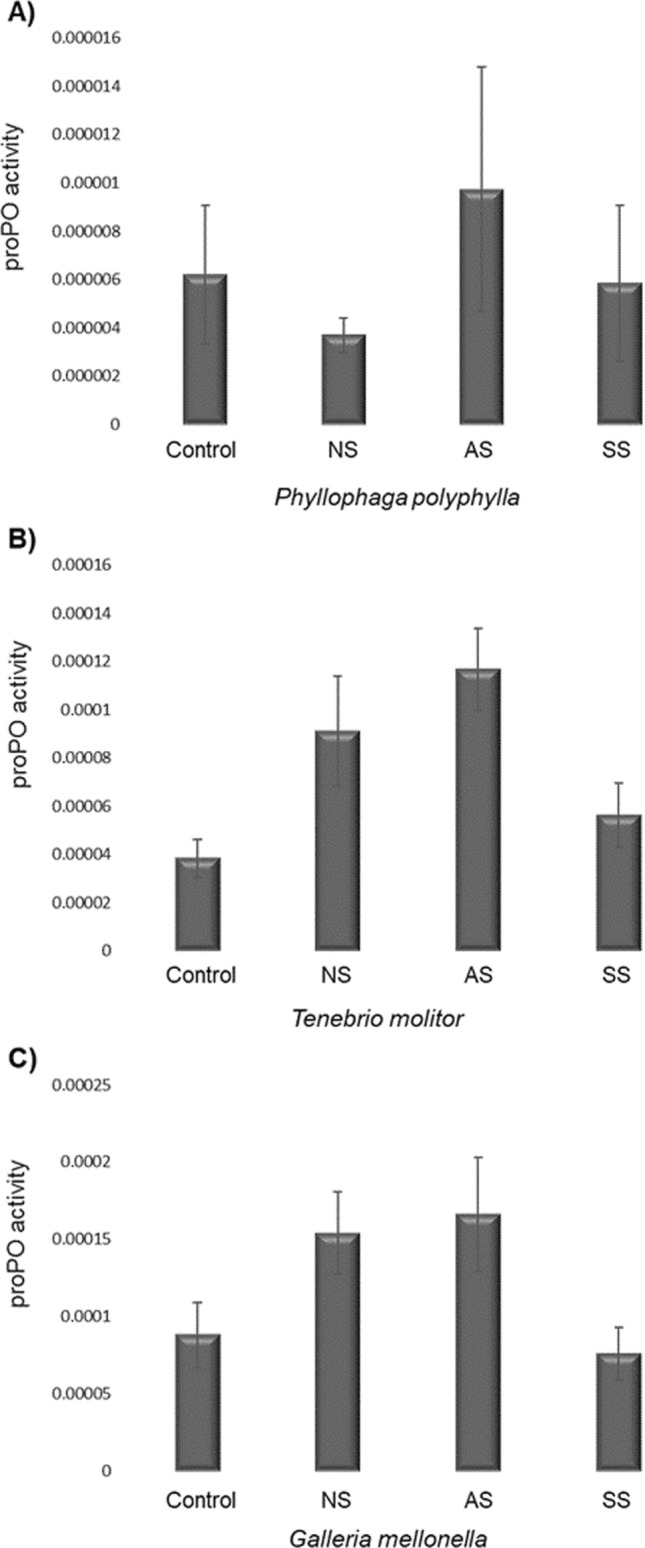
The proPO activity of insect larvae of *Phyllophaga polyphylla*, *Tenebrio molitor* or *Galleria mellonella* after injecting them with 1 dauer larvae of *R. regina* of the Natural Strain (NS; A), the Alternate Strain (AS; B) or the Saprophytic Strain (SS; C). In all cases, NS, AS and SS showed no statistical differences with the control group. See also the Table 1

The proPO activity showed significant differences according to Strain (*X*^*2*^ = 76.9, d.f. = 2, *p* = 0.001), Host (*X*^*2*^ = 19.16, d.f. = 3, *p* < 0.0001) and the interaction Strain*Host (*X*^*2*^ = 112.67, d.f. = 11, *p* < 0.001). The proPO of *P. polyphylla* showed no differences between the control group (Fig. 3A) and the groups NS (*p* = 0.9), AS (*p* = 0.9) or SS (*p* = 0.9). *T. molitor* did not show significant differences in proPO between the control group (Fig. 3B) and the groups NS (*p* = 0.9), AS (*p* = 0.16) or SS (*p* = 0.9). Finally, *G. mellonella* did not show significant differences between the control group (Fig. 3C) and the groups NS (*p* = 0.9), AS (*p* = 0.35) or SS (*p* = 0.9). Table 1 shows a resume of the results of proPO.

**Table 1:** Summary of results of *Phyllophaga polyphylla*, *Tenebrio molitor* or *Galleria mellonella* after injecting them with 1 dauer larvae of *R. regina* of the Natural Strain (NS; A), the Alternate Strain (AS; B) or the Saprophytic Strain (SS; C).

Immune response	Natural host			Alternate host			Saprophytic		
	*P. polyphylla*	*T. molitor*	*G. mellonella*	*P. polyphylla*	*T. molitor*	*G. mellonella*	*P. polyphylla*	*T. molitor*	*G. mellonella*
proPO	NS	NS	NS	NS	NS	NS	NS	NS	NS
PO	NS	NS	NS	NS	*▲*	*▲*	NS	NS	NS
Lytic activity	NS	NS	NS	NS	104775-153266NS	182130-165216NS	NS	NS	NS

*NS*  non-significant result (nematodes passed unnoticed by the immune response); *▲*  increased compared with control

### PO Activity

**Fig. 4:**
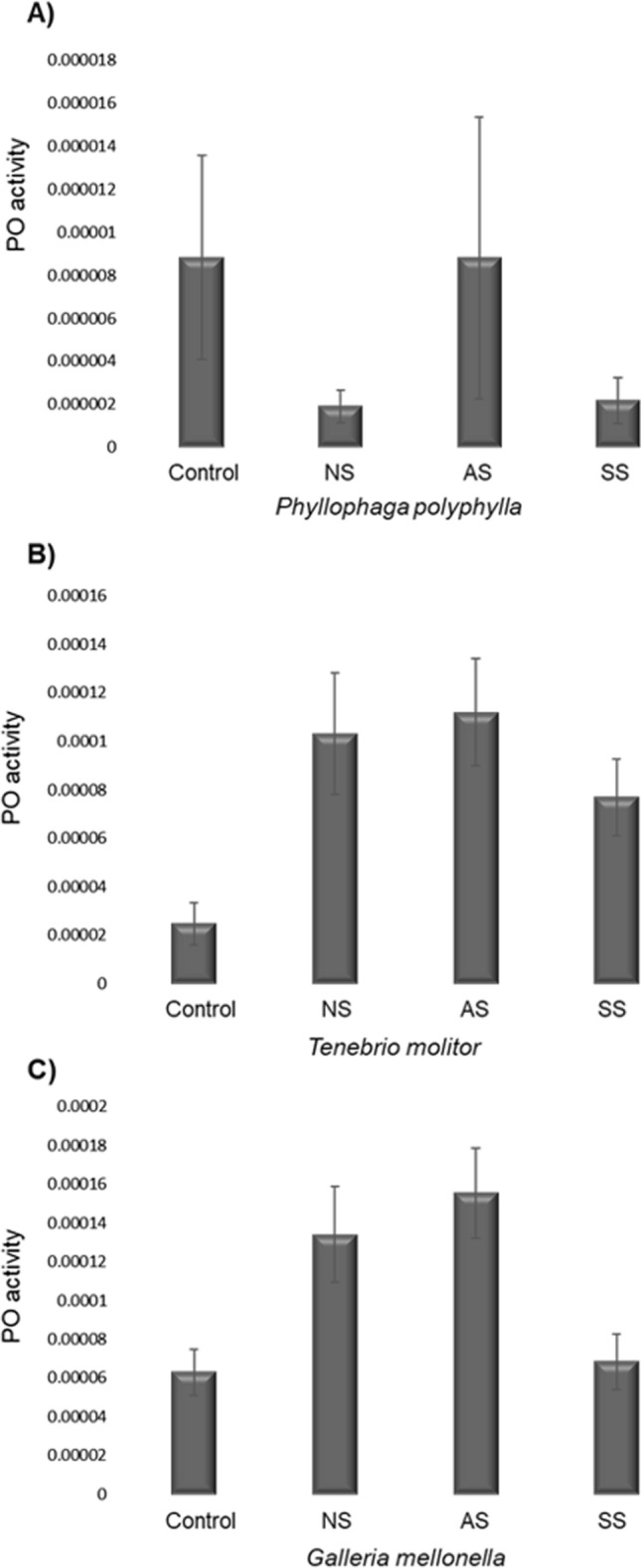
The PO activity of insect larvae of *Phyllophaga polyphylla*, *Tenebrio molitor* or *Galleria mellonella* after injecting them with 1 dauer larvae of *R. regina* of the Natural Strain (NS; A), the Alternate Strain (AS; B) or the Saprophytic Strain (SS; C). In most cases the PO activity of NS, AS and SS didn’t show statistical differences compared with controls. See also the Table 1

The PO activity showed significant differences according to Strain (*X*^*2*^ = 69.84, d.f. = 2, *p* < 0.0001), Host (*X*^*2*^ = 21.76, d.f. = 3, *p* < 0.0001) and the interaction Strain*Host (*X*^*2*^ = 114.28, d.f. = 11, *p* < 0.0001). The PO of *P. polyphylla* showed no differences between the control group (Fig. 4A) and the groups NS (*p* = 0.9), AS (*p* = 0.9) and SS (*p* = 0.9). Also, *T. molitor* did not show significant differences in PO between the control group and the groups NS (*p* = 0.9) and SS (*p* = 0.9). However, the group infected with AS was significant different than control group (*p* < 0.01; Fig. 4B). Finally, *G. mellonella* did not show significant differences between the control group (Fig. 4C) and the groups NS (*p* = 0.43) and SS (*p* = 0.9), but there were differences with the group AS (*p* < 0.03). On the other hand, the PO activity in the AS group was higher than the group NS (*p* < 0.02). Table 1 shows a summary of the results of PO.

### Lytic Activity

**Fig. 5:**
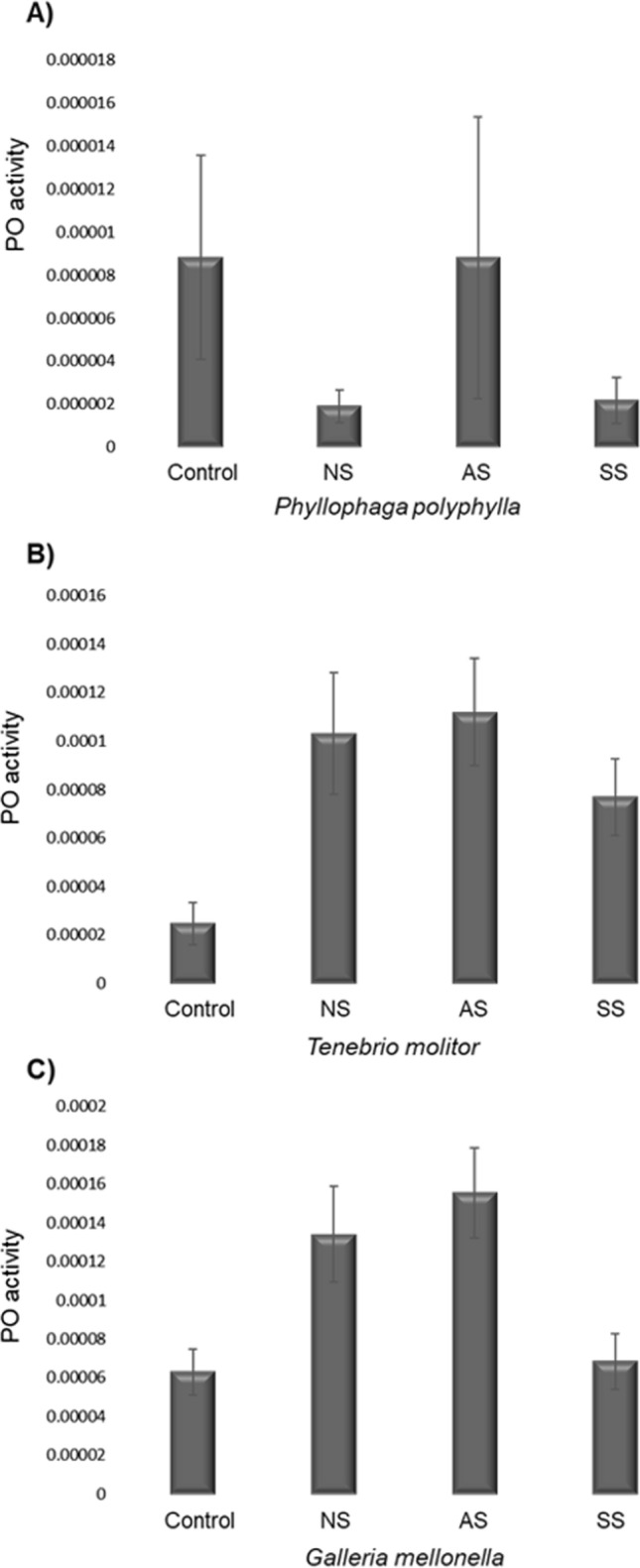
Lytic activity of insect larvae of *Phyllophaga polyphylla*, *Tenebrio molitor* or *Galleria mellonella* after injecting them with 1 dauer larvae of *R. regina* of the Natural Strain (NS; A), the Alternate Strain (AS; B) or the Saprophytic Strain (SS; C). The lytic activity of NS, AS and SS didn’t show statistical differences compared with controls. See also the Table 1

The lytic activity showed significant differences according to the Strain (*X*^*2*^ = 18.29, d.f. = 3, *p* =  < 0.001), Host (*X*^*2*^ = 29.92, d.f. = 2, *p* = 0.0001) and the interaction Strain*Host (*X*^*2*^ = 58.75, d.f. = 11, *p* < 0.0001). The lytic activity of *P. polyphylla* showed no differences between the control group (Fig. 5A) and the groups NS (*p* = 0.32), AS (*p* = 0.11) and SS (*p* = 0.9). *T. molitor* did not show significant differences in lytic activity between the control group and the groups NS (p = 0.9), AS (*p* = 0.76) and SS (*p* = 0.9). However, the lytic activity of group AS was higher than in the group NS (*p* < 0.01; Fig. 5B). Finally, *G. mellonella* did not show significant differences between the control (Fig. 5C), NS (*p* = 0.9), AS (*p* = 0.35) and SS (*p* = 0.9) groups. Table 1 shows a resume of the results of lytic activity.

## Discussion

The evolution of virulence was observed during four years with homologous (similar host ambient) and heterologous (dissimilar host ambient) lines of infection. More virulence was found with the homologous lines of infection: the NS strain against *P. polyphylla* and the AS strain against *T. molitor*. These combinations produced the most rapid death of the host, achieved by infection with a single nematode, but the heterologous challenge required ten nematodes to kill the host. The most significant virulence was detected among the heterologous lines against the lepidopteran *G. mellonella*.

In this and earlier studies, *R. regina* has been shown to infect different hosts species and therefore is considered a generalist [31, 47, 48]. The lifestyle of generalist parasites may imply evolutionary costs [37]. The current contribution demonstrated that higher doses of parasites were required to kill new versus natural hosts, which concurs with other reports [49, 50]. Overall, the dose of infection and the hosts' survival in homologous and heterologous lines of infection give support to two proposed concepts: the hosts belonging to the same taxonomic group as the natural host are susceptible to the same parasites [16], and the nature of such susceptibility may likely limit the expansion of parasites to new hosts [17, 51]. Phylogenetic remains associated with the host infection should maintain the parasite's latent capacity to be successful under new conditions if those traits are similar to its natural host [52]. Only particular parasite genotypes could successfully infect new hosts based on attributes that favor their survival and replication in a given time and place [14]. Overall, these results support the hypothesis of coincidental evolution of virulence and the short-sighted evolution of virulence [53]. The short-sighted evolution of virulence proposes specialization in a given microenvironment (i.e. the natural host), and therefore it is limited when a parasite encounters a new host [53]. This hypothesis has been validated by several studies finding lower virulence against new versus natural hosts (natural hosts being those with which the parasite coevolved; [13]. At the same time, this hypothesis demonstrates the limited scope of the phylogenetic relationship between the host species and their parasites. The Lepidoptera, compared with Coleoptera, died after a long time and with a higher dose.

For the strain breed on saprophytic medium (SS), attenuated virulence was found against *P. polyphylla* and *T. molitor*, perhaps due to the importance of live hosts as the causal factor of selective pressure during the evolution of virulence. However, the greater virulence against *G. mellonella* exhibited by nematodes cultivated in the saprophytic medium demonstrates the capacity of this environment to modify factors of virulence that favor an attack on the host, which corresponds to the hypothesis of coincidental evolution of virulence [14]. We did not evaluate the virulence factors that were favored or attenuated. Still, the greater virulence against *G. mellonella* produced by the saprophytic environment was likely due to the incorporation or loss of bacteria used by *R. regina* for feeding, considering the microbiota of this and other nematode species changes by its environment [30, 37]. For example, the SS strain but not the AS strain, is accompanied by bacteria of the *Brevundimonas, Bordetella, Myroides, Enterococcus, Bacillus, Clostridium, Actinomyces*, and *Goronia* genera [30]. It would be interesting to identify the bacteria that kill diverse hosts differentially and determine whether their attack on the host is based on distinct mechanisms.

The study of the immune response provides insights into how hosts defend themselves against infection and/or the strategies of the parasite for neutralizing these defenses. We found no significant differences in the immune response (proPO, PO and lytic activity) of the infected versus uninfected (control) hosts. Parasites were able to avoid detection by the host immune system. This is consistent with a previous study in which nematodes were unnoticed by the immune response [31], probably provided by molecular mimicry [16, 25], a strategy generalized for *R. regina* because it was found in homologous and heterologous challenges. Despite evidence of immune evasion, an increase was observed in the PO level against *T. molitor* and *G. mellonella* inoculated with the AS strain, revealing the activation of the PO system [54]. *Culex pipiens* (Diptera) and *Leptinotarsa decemlineata* (Coleoptera) are reported to activate PO and encapsulate *S. carpocapsae* [55, 56]. Since PO is directed against the nematode’s body to encapsulate it [20], a change of host could have a cost for *R. regina* in terms of molecular recognition. It is known that the immune response of insects can vary between species of nematodes and even between strains of the same species [15, 57]. For instance, the level of PO increases in *G. mellonella* (Lepidoptera) when infected with *H. bacteriophora*, but not when infected with *S. carpocapsae* or *S. glaseri* [26]. Once again, the current findings validate evasion of the host immune response [31], depending on the heterologous challenge in the parasite-host system. Therefore, the present data agree with the report of two strains of *S. glaseri* that cause distinct immune responses in the same host [37]. The nematodes’ cuticle is the main attribute that interacts with the insect’s immune system through passive and active mechanisms. The first one mimics or sequesters hemolymph components of the host to evade detection, whereas the second one actively destroys the immune effectors. Based on this, it seems that *R. regina* has a passive mechanism, but its details still need to be examined [31]. The role of the cuticle of nematodes in immune evasion deserves examination in a future study, given the key role of this component in the immune system of other species of nematodes [19, 20, 58]. Interestingly, *R. regina* did not appear to lose its capacity for immune evasion when confronting distinct microbiota, though the alternate niches provide different resources, both in quality and quantity. Since jumping hosts modify their virulence factors, there must be costs linked to the corresponding adaptation and should be tested further. We showed differences in microbiota between the insect-strain and saprophytic-strain at the beginning of the experiment [30]. Still, comparing the microbiota after experimental evolution is an ongoing test to know the further potential mechanism that might lead to the differences in the evolution of virulence.

Finally, the present results demonstrate that cultivating entomopathogenic nematodes in the lab can substantially alter their virulence potential, whether* in vivo* or* in vitro*. *G. mellonella* is commonly used to cultivate entomopathogenic nematodes of commercial importance [18]. Our results suggest that the virulence specificity may diminish parasites' effectiveness in controlling some plagues. For example, the cultivation of *Steinernema glaseri* in *G. mellonella* considerably reduces its virulence against *Pupil japonica*, its natural host [59].* In vitro* cultivation (in an artificial medium lacking live hosts), on the other hand, is herein shown to be a poor option for the massive production of nematodes [39, 60]. Indeed, it could lead to a significant decrease in virulence and, therefore, inefficiency in controlling insect plagues. It is crucial to analyze the virulence of entomopathogens from the point of view of the evolution of virulence to know potential scenarios that might limit or favor the attack on the host in natural conditions.

## Data Availability

Data will be available upon request.
